# The face-specific N170 component is modulated by emotional facial expression

**DOI:** 10.1186/1744-9081-3-7

**Published:** 2007-01-23

**Authors:** Vera C Blau, Urs Maurer, Nim Tottenham, Bruce D McCandliss

**Affiliations:** 1 Sackler Institute for Developmental Psychobiology, Weill Medical College of Cornell University, New York, USA; 2 Department of Cognitive Neuroscience, Faculty of Psychology, University of Maastricht, Maastricht, The Netherlands

## Abstract

**Background:**

According to the traditional two-stage model of face processing, the face-specific N170 event-related potential (ERP) is linked to structural encoding of face stimuli, whereas later ERP components are thought to reflect processing of facial affect. This view has recently been challenged by reports of N170 modulations by emotional facial expression. This study examines the time-course and topography of the influence of emotional expression on the N170 response to faces.

**Methods:**

Dense-array ERPs were recorded in response to a set (n = 16) of fear and neutral faces. Stimuli were normalized on dimensions of shape, size and luminance contrast distribution. To minimize task effects related to facial or emotional processing, facial stimuli were irrelevant to a primary task of learning associative pairings between a subsequently presented visual character and a spoken word.

**Results:**

N170 to faces showed a strong modulation by emotional facial expression. A split half analysis demonstrates that this effect was significant both early and late in the experiment and was therefore not associated with only the initial exposures of these stimuli, demonstrating a form of robustness against habituation. The effect of emotional modulation of the N170 to faces did not show significant interaction with the gender of the face stimulus, or hemisphere of recording sites. Subtracting the fear versus neutral topography provided a topography that itself was highly similar to the face N170.

**Conclusion:**

The face N170 response can be influenced by emotional expressions contained within facial stimuli. The topography of this effect is consistent with the notion that fear stimuli exaggerates the N170 response itself. This finding stands in contrast to previous models suggesting that N170 processes linked to structural analysis of faces precede analysis of emotional expression, and instead may reflect early top-down modulation from neural systems involved in rapid emotional processing.

## 1. Background

Adaptive behavior relies on the fast recognition of salient cues in the environment and facial expression is an important social index of potential threat [1]. Hence, face processing is commonly thought to be a rapid and highly automated process [2,3]. However, controversies exist concerning the role of emotional information during processing of faces, specifically the speed of encoding emotional information from faces and the relationship of such processing to the encoding of face structure.

Neuroimaging and neurophysiological evidence regarding face perception have often been used to argue that face processing is mediated by specialized brain systems [4,5]. Hemodynamic measures of brain responses have identified several brain regions that are consistently more activated for face stimuli compared to other object-categories, most notably the fusiform gyrus [6], ventral occipital and superior temporal cortex [7]. The existence of these specialized brain systems most likely reflects the salience of this stimulus-category in everyday life. Neuronal systems specialized for rapid perceptual analysis of emotionally salient events [8] consist of several brain structures that contribute to the recognition of emotional facial expression: the occipital cortices, amygdala, orbitofrontal cortex, basal ganglia, and right parietal areas, among others [9]. The human amygdala in particular has been shown to be a primary cortical structure for processing fear-eliciting stimuli [9-11]. This holds even when stimuli are presented below the threshold for conscious detection [12,13]. A subcortical mechanism involving the superior colliculus and pulvinar of the thalamus exists that provides input to the amygdala, suggesting a potential mechanism for residual visual processing of facial affect without awareness. Supporting evidence comes from subliminal studies on fear perception [12] as well as from studies of patients with striate lesions [14]. In addition to this subcortical route, monkey studies have revealed extensive reentrant connections from the amygdala to primary visual and ventral stream areas [15] indicating a possible cortical mechanism that mediates enhanced processing of threatening visual input. This is also in agreement with results from neuroimaging studies in humans [16]. These findings support the view that the amygdala is crucial for recognition of facial affect, can process such information rapidly, and in turn modulate activation in the ventral visual processing stream, even when stimuli are presented below the threshold for conscious detection [11].

In addition, a substantial amount of research has focused on the temporal dynamics of face processing using electrophysiological measures [17-19]. Intracranial event-related potentials (ERPs) reveal amplitude but not latency differences for face versus non-face objects reflected in an N200 potential recorded directly over ventral occipito-temporal cortex [18]. Similarly, recordings at the scalp demonstrate ERP components reflecting face-specific responses peaking at approximately 170 ms at occipital-temporal sites [19,20]. The N170 is dominant for faces [2] and eyes [21], and has shown substantial specificity for faces, typically demonstrating a smaller or absent N170 response for non-face stimuli [22]. The notion of specificity of visual processes involving face perception has been supported by several additional lines of evidence, including brain lesion data. Selective brain damage to aspects of the ventral pathway of the visual system can produce specific impairments in visual recognition of faces: a deficit termed face agnosia or prosopagnosia. Prosopagnosia is a specific deficit in the processing of faces that follows inferior occipito-temporal lesions [5]. The dissociation between damage to face processing without other visual deficits has been proposed by several researchers to suggest specific visual processes involved in structural analysis of face stimuli [18,23].

The time-course of analysis of facial stimuli has been the subject of several investigations that support a view of face recognition that involves at least two processing steps [24,25]. The first step has been linked to structural encoding of facial features. Additional processes associated with identity and emotional expression are performed later, as they may be thought to be dependent on the outflow of early structural encoding of raw featural information [3,26]. This idea dates back to a model of face recognition by Bruce and Young from 1986 [27], which describes the categorization of emotional expressions as resulting from the configuration of various facial features. This process is thought to depend on view-centered information, which is extracted during structural encoding of a face and provides the necessary raw visual input for the analysis of expression. Facial identity analysis, on the other hand, is described as a process requiring more abstract expression-independent information, which is again derived from the earlier view-centered analysis. View-centered and expression-independent descriptions together embody the structural encoding of a face. According to this functional model, initial structural encoding processes provide appropriate descriptive information for subsequent expression and recognition analysis.

In line with the early structural encoding operations described by Bruce and Young [27], early ERP components (e.g. N170) are thought to reflect rapid structural encoding of faces. Several lines of evidence support this conclusion. Bentin [19] for example showed that the N170 component is not exclusively found for upright faces, but also for inverted faces or isolated eyes. The authors conclude that the N170 is related to structural encoding processes and not identification.

Another line of evidence suggesting that the N170 reflects processes prior to face identification comes from studies demonstrating the absence of a familiarity effect on the N170 component [28,29]. For example, Bentin and Deouell [29] presented subjects with images of familiar faces, unfamiliar faces and butterflies with the instruction to monitor the screen for the appearance of butterflies. Faces elicited a clear N170 potential at posterior-temporal electrode sites that was significantly larger in amplitude than responses to the other stimuli. Furthermore, despite this sensitivity to the category of the stimulus class, N170 responses demonstrated no sensitivity to familiar versus unfamiliar faces. This result led Bentin and Deouell [29] to conclude that the N170, although specific for face processing, is not directly associated with face identification. Rather, it reflects a process that precedes identification, a process in line with the "structural encoding stage" suggested in the model of Bruce and Young [27].

Similarly, Eimer [28] demonstrated robust face-specific N170 responses, but such responses were completely insensitive to familiar vs. unfamiliar faces, even though such familiarity effects were evident later in the ERP response. Such results are entirely consistent with the stage-like model described above. Caharel and colleagues [30], however, have presented evidence challenging this view. Under some conditions of passive viewing, ERPs to unfamiliar faces demonstrated smaller N170 amplitudes than familiar faces.

Thus the literature on the modulation of the N170 by familiarity is mixed, and the factors that might lead a paradigm to be sensitive or insensitive to such modulation are yet to be fully explored. Nonetheless, the question of how the N170 component might be modulated by this and other types of information that might logically follow low-level structural analysis of faces is central to understanding the functional role of the N170 and its relation to other processes in models of face perception. Unfortunately the topography of this familiarity modulation is not well characterized, making it difficult to assess whether familiar minus unfamiliar N170 topographies implicate a broad negative shift across multiple channels, or whether the effect is a specific modulation of the channels specifically implicated in the N170 topography. Such topographic information may prove useful in distinguishing different underlying processes that contribute to this modulation. For example, Caharel and colleagues [30] suggest that the operations of structural encoding may intrinsically involve information concerning specific faces. An alternative account might involve other visual mechanisms that are sensitive to familiarity in a way that is not specific to faces, yet produce distinct and overlapping topographies within the time window of the face-specific N170. Additional studies using high-density channel arrays and investigating different task conditions may help shed light on such issues.

In addition to basic structural and familiarity information, facial stimuli also convey affective information. This type of facial information has also been studied in relation to the N170. Such research raises questions concerning how affective information processing is related to the idea proposed in the Bruce and Young [27] model that the face N170 reflects only the most basic structural encoding of facial information. In support of this model, there are multiple studies in the literature that have collected N170 responses to face stimuli that introduced experimental contrasts between classes of emotional expression, yet have found that despite sensitivity to the face N170, such responses were unmodulated by emotional affect [25,31-33].

Some studies have suggested that emotional content of stimuli are reflected primarily in later ERP components (e.g. P300) [26]. However, several reports from other paradigms demonstrate that simple affective modulation, such as classically conditioned aversion, can impact ERP components as early as 120–160 ms [34] suggesting the possibility that fast, emotion-sensitive processes may run in parallel to visual processes, and can have an impact on visual ERP responses.

Other reports reveal emotional effects that influence the face-specific N170 component [1]. Conversely, there is also evidence demonstrating that the N170 is entirely unaffected by emotional expression [25]. These discrepancies in experimental findings might be related to differences in design and stimuli. It is known that early visual evoked potentials are very sensitive to alterations in lower-level perceptual features, which poses a problem for many studies. In addition, it has been shown previously that repeated exposure to the same faces can lead to habituation effects attenuating neuronal responses to visual stimuli. Some authors [35] report stronger repetition suppression for fearful versus neutral faces in a recent fMRI study, indicating that habituation effects can potentially confound studies looking at emotional aspects of face processing.

Electrophysiological measures are a very powerful tool to examine the temporal dynamics underlying the different stages of face processing due to their high temporal resolution. However, they can only provide crude information about neural cortical sites for a particular cognitive task that is unquestionably inferior in comparison with modern hemodynamic techniques. Nonetheless, these measures provide the means to differentiate the time course of the N170 response to faces with different emotional expressions. Furthermore, electrophysiological measures permit one to examine whether structural and emotional aspects of face encoding are serial versus parallel processes as indicated by a modulation of the N170 response to faces.

The present study uses dense-array ERP recordings to investigate whether emotional expression effects are present in early visual ERP components including the face-specific N170. These set of analyses represent one set of questions in the context of a larger learning study. A separate analysis of this study investigates whether different cognitive processing strategies will affect subjects' learning performance in a paired associate learning task. In this task subjects had to attend to the association between a previously unknown orthographic character and a spoken word. The face stimulus always preceded the learning task making it possible to investigate the influence of emotional modulation of faces on the ERP signal independently of the learning task. Due to the complexity of the task, the present article will focus on the ERP results associated only with face processing and their modulation by expression. The results related to the influence of cognitive strategies on paired associate learning are discussed elsewhere [36]. For the purposes of the present study, male and female faces with either neutral or fearful expressions were presented as irrelevant stimuli preceding trials in a demanding associative learning task. Explicit task demands were arranged to avoid differentially biasing subjects attention toward encoding face structure or emotional content [31,37]. This design follows from the suggestion that emotional modulation of early ERP responses may be most likely when those stimuli appear as incidental to the primary task [38,39]. Finally, stimuli were created with large sets of faces, and sufficient numbers of trials to permit analysis of face N170 and emotional effects in the first and last half of the experiment.

According to the electrophysiological and functional neuroimaging results reviewed above, we hypothesized that if encoding of structural and emotional expression are parallel processes such that fast emotional encoding modulates the N170 response, the N170 component will show an amplitude difference for fearful versus neutral faces. Alternatively, if early structural encoding of faces and analysis of emotional affect are serial processes, and emotional encoding necessarily follows structural encoding of faces, then the N170 component for faces will demonstrate no amplitude differences for fearful versus neutral stimuli. Furthermore, if emotional modulation effects are temporally coincident with N170 responses to faces, the topography of this emotional effect can be investigated to examine whether this effect might be characterized as a modulation of the N170, in which the fear minus neutral face N170 would produce a topography that itself resembles a face N170 topography.

## 2. Methods

### 2.1 Participants

Thirty-four healthy participants were recruited by advertisement and paid for their participation. The sample comprised 15 men and 19 women between ages 18 to 36 years of age. Inclusion criteria were English as first language, predominant right-handedness, and normal or corrected-to-normal vision. Exclusion criteria were of two kinds: behavioral and electrophysiological. Behavioral exclusion was determined by history of language disorders as assessed by the TOWRE test of word reading efficacy (1.5 SD around the mean for chronological age [40]; and a response measure of associative learning performance as indicated by 80% or more correct answers on trained items). Electrophysiological exclusion was determined by data quality (30% or more rejected trials per experimental condition). Out of thirty-four initial participants, eight had to be excluded due to low performance in the primary task, and two due to technical problems and resulting low ERP data quality. Participation was entirely voluntarily and in accordance with the National Health and Medical Research Council ethical guidelines.

### 2.2 Stimuli

**Figure 1:**
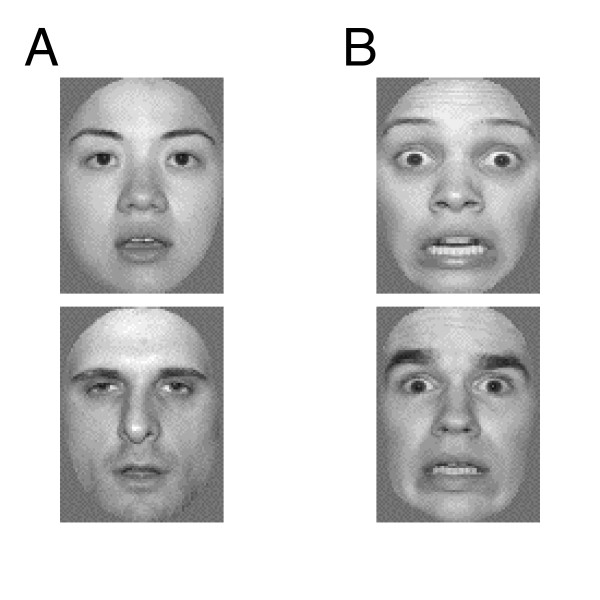
Sample stimuli used to elicit face N170 responses in (A) the neutral condition, and (B) the fear condition.

Face stimuli were 16 high-resolution photographs, half depicting a neutral ("calm") expression and the other half depicting a fearful expression. Stimuli were selected from the NimStim Set of Facial Expressions [41], which is a standardized database of photographs of professional actors portraying various emotions. All images were matched for luminance and contrast and subtended a horizontal visual angle of 2° and a vertical angle of 3°. In addition, all face stimuli were converted to grayscale, brought into an oval shape to remove hair, neck, and background information and were then presented on a light grey background (figure 1). Additional stimuli, which were incidental to the current study, included orthographic stimuli consisting of 16 black line-drawing characters on a white background and 16 audio files of spoken English words.

### 2.3 Design and procedure

After written consent was obtained to participate in an experimental protocol approved by Cornell University Medical College, participants were asked to rate all 16 faces in terms of perceived intensity of their emotional expression. Participants were then seated within a small, electrically shielded, dimly lit room. Visual images were presented on a computer screen (refresh rate, 60 Hz), which was situated at 100 cm from the participants' eyes. All subjects received the instruction to maintain central eye fixation and were observed via a monitor located outside the EEG-chamber in a control room.

### 2.4 Intensity ratings for face stimuli

Prior to EEG recording, participants were asked to rate all 16 face stimuli on perceived intensity of their emotional expression on a scale from 1 to 5.

### 2.5 Primary task instructions

Participants were told that on each trial they would see a face, followed by a line drawing, followed by an auditory word, that the task was to learn the associations between the line drawing and the auditory word, and that the face stimulus was completely irrelevant. The primary task phase of the experiment was ~ 20 minutes long, divided into 4 blocks. The subjects' primary task required them to memorize the association between a novel orthographic character and its corresponding spoken counterpart [36], which included a between-subjects manipulation regarding the learning strategy they should employ. Half the subjects were instructed to encode the symbol and its association to the subsequent spoken word by using a holistic whole word encoding strategy (Group 1) while the other subjects were instructed to follow a grapheme-phoneme mapping strategy (Group 2). Regardless of which instruction condition subjects were assigned to, the trial structure was the same with regard to the incidental nature of the face stimuli, the timing of the trials, and the learning materials. Stimuli were presented in simple sequences during the entire experiment. The interval between the face stimulus (duration 100 ms) and the following visual stimulus was 200 ms. The stimuli of the primary task were presented after a gap of 200 ms consisting of a visual symbol presented for 2300 ms and a spoken word presented for 600 ms.

This primary task phase of the study consisted of 320 trials, which included 16 individual face stimuli (8 neutral, 8 fearful) presented 20 times each in random order with the constraint that each of the 16 faces were presented across each of 16 contiguous trials.

### 2.6 Primary task learning assessment

Participants' learning of the associations between symbols and spoken words in the primary task was tested via a subsequent symbol-to-sound matching task. The presentation of each symbol was followed 600 ms later by an auditory presentation of a spoken word. Half the trials contained a match, in which the visual symbol and spoken word corresponded to pairs presented in the primary task. The other half of the trials consisted of distractors, in which the symbol and spoken word did not match. Spoken words for distractor trials were selected from the primary task, and thus were equally familiar as the spoken words in the match trials. No faces were shown in this task, as the purpose was to assess compliance in the primary task. Participants were asked to indicate via button press whether the symbol and the sound matched based on their learning in the preceding primary task., Subjects were given a maximum of 2000 ms to respond following the onset of the spoken word. This part of the experiment consisted of three blocks and 128 trials per condition. These data provide a rough characterization of the level of engagement in the primary task, as well as an opportunity to examine the possibility of emotional modulation effects on learning robust enough to impact later recall.

### 2.7 EEG recording

Presentation of stimuli was controlled via a PC running E-Prime experimental programming software [42]. The 128-channel dense array EEG was recorded using the Geodesic Sensor Net [43]. EEG was sampled at 250 Hz with the vertex electrode as the online reference. Gain and zero calibration were performed prior to the start of every recording and impedances for all channels were kept below 50 kΩ.

### 2.8 Electrophysiological analysis

**Figure 2:**
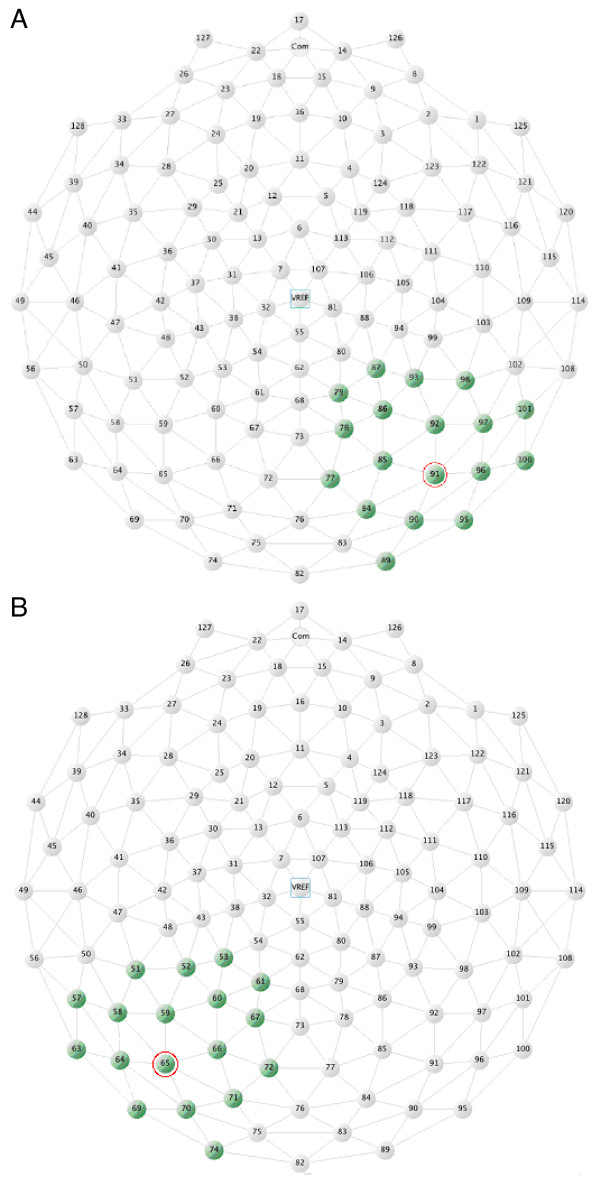
Polar presentation of channel locations in the 128 channel Geodesic sensor net. The 18 occipital-temporal channels used in the statistical analyses are highlighted in green for (A) the right hemisphere, and (B) the left hemisphere. Channels used to illustrate the time-course of the ERP effect (see Figure 3) are indicated in red.

EEG data were analyzed using the EGI analysis tool [43]. A 0.1 high pass filter was applied online. Subsequent to recording, raw data were filtered using a 60 Hz notch filter in order to remove predominant noise related to electrical equipment. ERP data were computed using a 1000 ms epoch (100 ms prestimulus, 900 ms poststimulus) and time locked to the onset of the face stimulus. Artifact Rejection Criteria were defined as 70 μV for vertical eye movements, whereas horizontal eye-movements were negligible, so no correction was applied. A recording segment was marked bad if it contained more than 10 bad channels. A channel was marked bad in one of three situations: 1. when the fast average amplitude exceeded 100 μV; 2. the differential average amplitude exceeded 50 μV; or 3. the channel showed zero variance. In addition, channels were excluded that contained more than 20% bad segments across the entire recording and interpolated using spherical spline interpolation including all channels of the spherical surface of the head. ERP data were then averaged, corrected for a 120 ms baseline and re-referenced to an average of all 128 electrodes. Two relevant event-categories were used to compute the single-subject average: the neutral condition and the fear condition. Additional analysis investigated whether both N170 and emotional modulation effects might appear equally robust across the first 80 trials (40 fear and 40 neutral trials) as compared to the last 80 trials. Mean ERPs were computed across subjects and visualized topographically in Net Station. For statistical extraction an adaptive mean amplitude value was used and the extraction was limited to 18 occipital-temporal channels in each hemisphere. Extraction of amplitude values was centered around the mean latency of the face specific N170 component (130–210 ms poststimulus). Figure 2 outlines the groups of channels used for extraction of N170 mean amplitude values. The channels indicated in red were solely used for visualization.

Data were analyzed using a repeated measures analysis of variance (ANOVA) with factors Emotion, Time of presentation (i.e. first half, second half), Hemisphere, Group (i.e. strategy instruction), and Gender of presented face.

## 3. Results

### 3.1 Pre-recording intensity ratings of facial affect

As expected, subjects perceived the fear faces as more intense in their emotional expression compared to neutral faces (mean fear: 4.00, SD: .54; mean neutral: 2.48, SD: .46). This difference was significant (t (23) = 13.245, p < .001) even when breaking the contrast down across the two instructional groups separately (group1: fear: 3.85, SD: .57; neutral: 2.35, SD: .42; t (23) = 8.210, p < .001) (group2: fear: 4.15, SD: .50; neutral: 2.62, SD: .48; t (23) = 10.483, p < .001). An analysis on gender effects revealed no significant difference for the perception of fear (female: 4.02, SD: .57; male: 3.97, SD: .54), but showed that females overall rated neutral faces as more intense in their emotional expression than male participants (female: 2.69, SD: .44; male: 2.21, SD: .32; F (1, 23) = 8.142, p < .05).

### 3.2 Primary task assessment

Assessment of performance in the subsequent audio-visual character matching task ensured that participants engaged in the primary task. Mean accuracy was 92.5% (SD: .20) with a mean reaction time of 986 ms (SD: 158.50). No significant effects on accuracy or reaction time were found for items paired with fear faces during the training period as compared to items that were paired with neutral faces.

### 3.3 N170 amplitude

**Figure 3:**
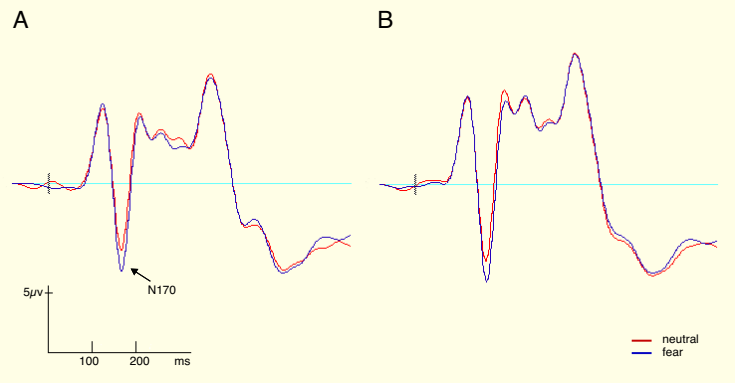
Timecourse (in milliseconds) of the ERP face response (in microvolts) for the fear and neutral facial affect conditions, diplayed for (A) left occipital-temporal channel 65, and (B) right occipital-temporal channel 91.

As predicted, a large negative component was observed in response to faces at ~ 170 ms post-stimulus at occipital-temporal electrodes. No evidence of earlier effects was present. Channel 69 in the left hemisphere together with channel 95 as its right-hemispheric complement were selected based on peak negative amplitude out of 18 occipital-temporal channels (polar projections of the channel locations are shown in figure 2). Figure 3 illustrates the grandmean waveforms for 69 and 95 at inferior occipito-temporal electrode sites demonstrating that N170 responses to faces were enhanced for fear faces relative to neutral faces.

### 3.4 N170 topographies

**Figure 4:**
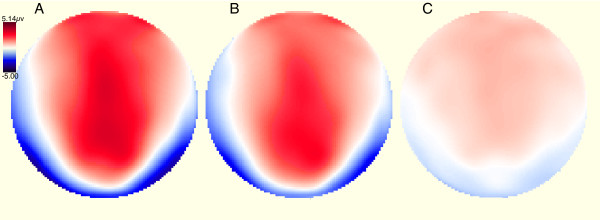
Polar projections of spherical spline interpolations of the voltage topography (in microvolts) 170 milliseconds following the onset of the face stimulus for three conditions: (A) fear, (B) neutral, (C) fear minus neutral.

The N170 topographic maps for both conditions revealed an enlarged negativity over occipital-temporal electrode sites along with a positive dominance over fronto-central channels (Fig. 4). Figure 4 also shows the difference-topography created by subtracting the neutral from the fear condition. As can be seen in this graph, the typical N170 topography that was observed in each of the two emotion-conditions is preserved when looking at the difference map.

Amplitude data were analyzed using a repeated measures ANOVA. A significant greater negativity was observed to fear faces as compared to neutral faces (F (1, 23) = 26.69, p < .05). No interaction with hemisphere, 'time of presentation', gender or instruction group was present. Means and standard deviations for the different conditions are reported in Table 1.

**Table 1:** N170 Amplitudes (in μV) for Emotion, Time of Presentation, Hemisphere, Group and Gender.

	**Fear**							
	
	**1^st ^half**				**2^nd ^half**			
	
	**LH**		**RH**		**LH**		**RH**	
	Mean	SD	Mean	SD	Mean	SD	Mean	SD

**Group**								
1	-4.397	2.309	-4.703	3.438	-4.060	2.115	-4.316	3.005
2	-5.632	5.832	-5.317	5.999	-5.403	5.427	-5.136	5.687
**Gender**								
Female	-4.261	2.694	-4.546	2.645	-4.033	3.066	-4.174	3.057
Male	-6.069	6.030	-5.660	6.911	-5.710	5.221	-5.637	6.001

	**Neutral**							
	
	**1^st ^half**				**2^nd ^half**			
	
	**LH**		**RH**		**LH**		**RH**	

	Mean	SD	Mean	SD	Mean	SD	Mean	SD

**Group**								
1	-2.902	2.407	-3.351	3.215	-3.179	2.250	-3.503	2.934
2	-4.942	5.473	-4.507	5.569	-4.027	5.153	-3.766	5.366
**Gender**								
Female	-3.424	2.776	-3.621	3.052	-3.022	3.093	-3.428	3.104
Male	-4.620	5.865	-4.361	6.133	-4.416	4.904	-3.924	5.629

Group 1 received Whole Word strategy instructions; Group 2 received Grapheme-Phoneme strategy instructions, but were otherwise identical in all the procedures.

### 3.5 N170 latency

The N170 peak in response to face stimuli was centered around ~ 170 ms post-stimulus at occipito-temporal channels. N170 mean latency for the fear condition was 171.5 ms (SD: 16.41 ms) and 170.4 ms (SD: 18.52 ms) for the neutral condition. Two-way ANOVAs revealed no significant difference in latency of N170 for fear versus neutral faces, yet showed a main effect of hemisphere. N170 latency (F (1) = 6.152, p < .05) was significantly earlier in the right hemisphere (right hemisphere mean: 168.49 ms; SD: 3.30 ms; left hemisphere mean: 173.44 ms; SD: 3.77 ms; F (1, 23) = 6.152, p < .05). No interaction effects were observed.

## 4. Discussion

The objective of the present study was to investigate whether the N170 ERP component is sensitive to emotional facial expressions in addition to playing a role in structural encoding of faces. According to the two-stage model of face processing the face-specific N170 ERP component is linked to structural encoding of faces [3,25,28]. However, this view has been challenged by reports suggesting that the N170 is not strictly sensitive to structural features of faces [1,26,34] but is also modulated by emotional facial expression. The present findings support this conclusion. An N170 component was elicited at occipital-temporal electrode sites both for fear and neutral faces. Importantly, N170 responses to fear faces were significantly larger in amplitude than N170 responses to neutral stimuli independent of other factors including hemisphere, gender of the actor, or instructional group, and the N170 and emotional effects were equivalent in the first and second halves of the experiment.

These findings are in line with results from other recent studies. Modulation of an early posterior negativity has been demonstrated in response to various emotional stimuli including threatening images [44]. Another recent ERP study [26] also demonstrated that early N170 responses are differentially affected by emotional facial expression. However, in contrast to the results reported here, these authors report N170 emotional modulations only for inverted faces and not for upright faces, suggesting that subjects may have been processing the inverted faces in a more part-based fashion relative to upright faces.

This raises questions about the particular conditions of the present study that might have been conducive to eliciting rapid emotional effects on upright faces that were concurrent with the time-window of the N170. Although the current experiment was not initially designed to examine such a hypothesis with explicit experimental contrasts, it is potentially useful to review certain design elements of this study that might have contributed to eliciting such effects. Participants in this particular study were not engaged in a perceptually demanding primary task, freeing them to view the face stimuli as incidental to their primary task. Thus, although subjects most likely directed spatial attention towards the faces, the task-conditions made the face stimuli an irrelevant aspect of the task. In fact, most of the subjects reported later on that they could easily ignore the faces, as instructed. Recent studies have suggested that such incidental presentation of faces may play a pivotal role in the presence or absence of emotional modulation of N170 responses [38,45]. Similarly, Batty and Taylor [1] presented subjects with photographs of faces (six basic emotions plus neutral baseline) under conditions that required them to focus on a primary non-face task and make responses to non-face objects. These authors also found that the amplitude of N170 in response to fearful faces was larger than to neutral or surprised faces, suggesting an emotional modulation concurrent with the N170. The present study did not include a 'focal-attention' condition for comparison, so no strong conclusion about the role of attention can be drawn here. However, the present findings indicate that even under task conditions in which faces are unrelated to the task, sensitivity of the N170 component to emotional information exists. Additional research that introduces explicit attention contrasts will be necessary to determine whether incidental presentation of stimuli directly modulates the presence or absence of emotional modulation of the N170.

An additional potential difference between this study and previous studies that have not found emotional modulation of N170 responses may lie in the stimulus set. In contrast to previous studies, stimuli in the present study were cropped to remove hair, neck, ear, and background information that might detract from facial expression processing. Furthermore, these stimuli were normalized in size, brightness and contrast using a histogram-based method for equalizing perceptual variations. It is likely that in doing so, the current study removed several irrelevant sources of variance (i.e. increasing the homogeneity of the stimulus set) and reduced factors that might lead to a loss of sensitivity to emotional stimuli (i.e. few number of faces with high number of repetition), thereby increasing the salience of the emotional expression information relative to these other forms of irrelevant information.

Moreover, the electrophysiological effects obtained may also be linked to the large stimulus set employed. The present design made use of eight different faces per emotional condition, which might have worked against potential habituation effects. As mentioned previously, Ishai and colleagues [35] reported stronger repetition suppression for fearful versus neutral faces in a recent fMRI study, indicating that habituation effects can potentially confound studies looking at emotional aspects of face processing. For this reason it is argued that preventing potential familiarity/fatigue effects over the course of the experiment might be critical for studies investigating early visual effects of emotion. Furthermore, the current design alternated facial stimuli with another class of visual stimuli (i.e. the novel visual symbols), which likely served to prevent habituation. This notion is consistent with a recent study [46] that directly contrasted the amplitude of the N170 to faces within presentation blocks that contained only face stimuli versus presentation blocks that alternated between faces and visual symbols, while controlling for interstimulus intervals between faces. N170 responses to pure face blocks were largely attenuated relative to N170 responses to the same faces when they were interspersed with visual symbols. This may suggest that designs that present faces without intervening classes of stimuli may lead to smaller, habituated face N170 responses, which may reduce sensitivity to emotional modulation effects.

The finding of an N170 modulation by emotional facial expression was further validated by establishing this effect separately within the first and second halves of the experiment. Furthermore, results even remained significant when looking at each of the hemispheres or instructional groups separately. Thus, even when testing the present hypotheses with reduced power, the results remain to show robust main effects of emotional condition. Therefore, the current findings are in line with the general conclusions of a valence-dependent processing mechanism that is reflected in N170 ERP amplitude.

No differences in N170 latency were observed for fear versus neutral faces, a finding that is consistent within the literature. Although it is known that the face specific N170 component can be delayed by inversion of faces [47], several studies that have systematically modulated facial expression either do not report N170 emotion effects [28] or report that effects are restricted to ERP amplitude [39].

As noted earlier, some have argued that face stimuli are first analyzed on a structural level before emotional significance can be assigned [25], and that early visual components (the N170 in particular) have been thought to reflect rapid structural encoding of faces, while later components (e.g. P300) more likely reflect categorization of motivationally relevant information and the allocation of attention to emotional content of stimuli [26]. The present findings do not support this characterization, but rather argue in favor of a fast parallel system for emotional effects that are not sequentially dependent on the structural facial processes associated with the N170, and further suggest that the face N170 may be modulated by emotional processes. In this regard, the present study extends earlier findings [1] by demonstrating that the topography of the emotional modulation (fear versus neutral) is highly similar to the overall N170 topography. This provides support for the notion that emotional valence of a stimulus, perhaps via a fast parallel system sensitive to such information, serves to modulate the process of the N170.

This proposal is consistent with reports from neuroimaging studies in humans [11,16] that demonstrate specialized neuronal systems associated with rapid perceptual analysis of emotionally salient events, such as fearful facial expressions [8,48]. The amygdala is considered a primary structure for processing fear stimuli [10,49]. Non-human primate studies have demonstrated significant feedback connections between the amygdala and visual areas within occipital cortex [50], providing a possible cortical mechanism that mediates enhanced processing of threatening visual input. The present findings support this view in the sense that they point to the need for a neuronal, cortical mechanism that modulates typical visual analysis of face information (as indexed by the N170) in the presence of emotional information.

It is important to stress, however, that the current findings provide no direct means of assessing the precisely localized neural sources of the emotional effect, the face N170 effect, nor the modulation of the face N170 by emotion. Although the topographic findings are suggestive, they also are consistent with a large and rather unconstrained set of potential neural sources. Additionally, it is also possible to attempt to account for the present findings by speculating that underlying structural descriptions associated with the N170 are sufficiently detailed to drive the observed emotional expression effects on the face N170. Additional research designed to further explore the potential relationship between amygdala and visual regions will likely benefit from the use of a wider variety of expressions known to modulate amygdala activity, as well of source localization techniques to examine the potential neural sources of the emotion and face effects. Nevertheless, the current findings provide timecourse and topography evidence that is inconsistent with a serial process account by which encoding the structural information of faces associated with the N170 precedes evaluation of emotional expression. Furthermore, it is suggested that some aspects of the current experiment, such as the presentation of facial stimuli as incidental to the primary task, the use a broad range of stimuli manipulated to minimize the impact of non-facial information, and the way in which facial stimuli were alternated with another class of stimulus, may have contributed to the sensitivity of this paradigm to the observed effects.

## 5. Conclusion

This work demonstrates that early electrophysiological responses to faces are enhanced for emotional as compared to neutral stimuli, at least under non-explicit task conditions. In particular, this emotional effect is reflected in an early modulation of the N170 face-specific ERP component. Contrary to the two-stage model of face processing, face structure and emotion produce ERP responses within the same N170 time-frame. Moreover, difference waves between fear and neutral faces demonstrate a time-course and topography very similar to the N170 topography typically elicited by faces. These findings are more consistent with accounts that propose that the face-specific N170 is modulated by a rapid, parallel encoding system sensitive to emotional expressions of faces [8].

## Authors' contributions

VB participated in the initial design of the experiment, programmed the experiment, collected a majority of the data, performed the majority of the analyses, and drafted the initial paper. UM provided consultation in experimental design and analysis, assisted in data collection, and contributions to the final draft. NT created the face stimulus database, and provided consultation on the final draft of the paper. BDM, the principal investigator of the overall research project, contributed the initial conception for the study, provided oversight and consultation for each phase of the study, and completed the final draft. All authors read and approved the final manuscript.
